# Identification and characterization of a novel 43-bp deletion mutation of the *ATP7B* gene in a Chinese patient with Wilson’s disease: a case report

**DOI:** 10.1186/s12881-018-0567-z

**Published:** 2018-04-12

**Authors:** Gang Liu, Dingyuan Ma, Jian Cheng, Jingjing Zhang, Chunyu Luo, Yun Sun, Ping Hu, Yuguo Wang, Tao Jiang, Zhengfeng Xu

**Affiliations:** State key Laboratory of Reproductive Medicine, Department of Prenatal Diagnosis, The Affiliated Obstetrics and Gynecology Hospital of Nanjing Medical University, Nanjing Maternity and Child Health Care Hospital, No.123, Tianfeixiang, Mochou Road, Nanjing, 210004 Jiangsu Province China

**Keywords:** Wilson’s disease, *ATP7B*, Novel mutation, FoSTeS/MMBIR

## Abstract

**Background:**

Wilson’s disease (WD) is an autosomal recessive disorder characterized by copper accumulation. *ATP7B* gene mutations lead to ATP7B protein dysfunction, which in turn causes Wilson’s disease.

**Case presentation:**

We describe a male case of Wilson’s disease diagnosed at 10 years after routine biochemical test that showed low serum ceruloplasmin levels and Kayser–Fleischer rings in both corneas. Analysis of the *ATP7B* gene revealed compound heterozygous mutations in the proband, including the reported c.3517G > A mutation and a novel c.532_574del mutation. The c.532_574del mutation covered a 43-bp region in exon 2, and resulted in a frameshift mutation (p.Leu178PhefsX10). By base sequence analysis, two microhomologies (TCTCA) were observed on both deletion breakpoints in the *ATP7B* gene. Meanwhile, the presence of some sequence motifs associated with DNA breakage near the deletion region promoted DNA strand break.

**Conclusions:**

By comparison, a replication-based mechanism named fork stalling and template switching/ microhomology-mediated break-induced replication (FoSTeS/MMBIR) was used to explain the formation of this novel deletion mutation.

**Electronic supplementary material:**

The online version of this article (10.1186/s12881-018-0567-z) contains supplementary material, which is available to authorized users.

## Background

Wilson’s disease (WD, OMIM #277900), an autosomal recessive disorder characterized by abnormal copper accumulation and related toxicities, is caused by mutations in the *ATP7B* gene (OMIM *606882) [1]. The *ATP7B* gene is located on 13q14.3 spanning ~ 100 kb, including 21 exons and 20 introns; it encodes a transmembrane copper-transporting P-type ATPase of 1465 amino acids, which plays a crucial role in maintaining body copper homeostasis and is involved in copper transport into the plasma by ceruloplasmin as well as copper excretion from the liver [2–4]. *ATP7B* gene mutations lead to ATP7B protein dysfunction, which in turn causes accumulation of copper in the liver, brain, kidneys and corneas, with a wide range of clinical symptoms, including hepatic disorders, neuronal degeneration of the brain, and Kayser-Fleischer rings at the corneal limbus [5, 6]. The prevalence of WD is estimated at 1 in 30,000, and the heterozygous carrier rate approximates 1 in 90 in many populations [7, 8]. Without timely diagnosis and treatment, WD can be fatal [9]. Early detection and intervention is critical in preventing disease progression and irreversible sequelae. Genetic testing for the detection of biallelic *ATP7B* mutations do not only help diagnose WD, but can be also used for prenatal screening. About 540 disease-causing variants have been reported in *ATP7B* according to the HUGO database, and are mostly point mutations and small insertions or deletions distributed in all exons/introns (http://www.wilsondisease.med.ualberta.ca/database.asp). Screening of all exons and flanking intronic regions for *ATP7B* mutations is usually performed by direct sequencing, e.g. Sanger sequencing and next-generation high-throughput sequencing. However, mutations are identified in only one allele or none in a substantial number of WD patients [10]. A possible reason could be that partial gene deletions of one or more exons are not detected by current methods, for example c.4021 + 87_4125-2del2144, c.51 + 384_1708-953del8798, and c.52-2671_368del3039 of the *ATP7B* gene [11–13]. For unexplained WD cases, in whom no or one allele is mutated, detecting partial or whole gene deletions/duplications by MLPA assay, QPCR, and other methods is required. Here we report a novel 43-bp deletion by direct Sanger sequencing of all exons in the *ATP7B* gene, which is by far the biggest exonic deletion within a single exon of the *ATP7B* gene detected in a WD patient. Meanwhile, we comprehensively analyzed the possible mechanisms underlying the formation of this deletion mutation, and a replication-based mechanism named fork stalling and template switching/microhomology-mediated break-induced replication (FoSTeS/MMBIR) seems to explain it clearly [14–16].

## Case presentation

The proband was the first child of healthy non-consanguineous Chinese parents from Jiangsu Province. He was born at 39 weeks of gestation, and the perinatal period was normal. He was referred to our hospital at the age of 10, for paroxysmal headache of an approximate one-month duration. Paroxysmal forehead pain in this patient consisted of daily episodes at different times, even up to dozens of times, automatically disappearing after 1–2 min. Before the onset of these symptoms, his developmental milestones were normal. Physical examination was normal. Neurological examination revealed clear consciousness, normal language use, normal muscle tone and tendon reflexes of the limbs, and normal gait. However, he presented with rapid alternating movements of slightly clumsy hands and slightly unstable finger-to-nose movements. Blood routine examination for red blood cell count, white blood cell count, platelet count, and hemoglobin was normal. Serum total bilirubin, direct bilirubin, total bile acids albumin and plasma ammonia levels were in control ranges, as well as serum alanine transaminase and aspartate aminotransferase. Normal serum total cholesterol, triglyceride, plasma glucose and homocysteine levels were observed. Serum creatinine and blood urea nitrogen were normal, as well as serum electrolytes, including potassium, sodium and chloride. Serum ceruloplasmin and copper levels were 20.6 mg/L (normal 200~ 600 mg/L) and 1.53 μmol/L (normal 11–22 μmol/L). Kayser–Fleischer rings in both corneas were observed under slit-lamp. Abdominal ultrasonography showed mild spleen enlargement and slightly increased reflectiveness of the liver and both kidneys, but no hepatomegaly. Cranial magnetic resonance imaging (MRI) showed a decreased signal intensity in T1WI and an increased signal intensity in T2WI in bilateral caudate nucleus. The diagnosis of WD was clinically made based on low serum ceruloplasmin levels and Kayser–Fleischer rings, with the Wilson’s disease scoring system applied as WD diagnostic criteria [17]. Improvement was observed after 4 weeks of treatment with D-penicillamine in another hospital. To further confirm the diagnosis of WD, DNA sequence analysis of the *ATP7B* gene and bioinformatics analysis were performed by the methods in Additional file 1. The CARE guidelines were followed in reporting this case.

**Fig. 1:**
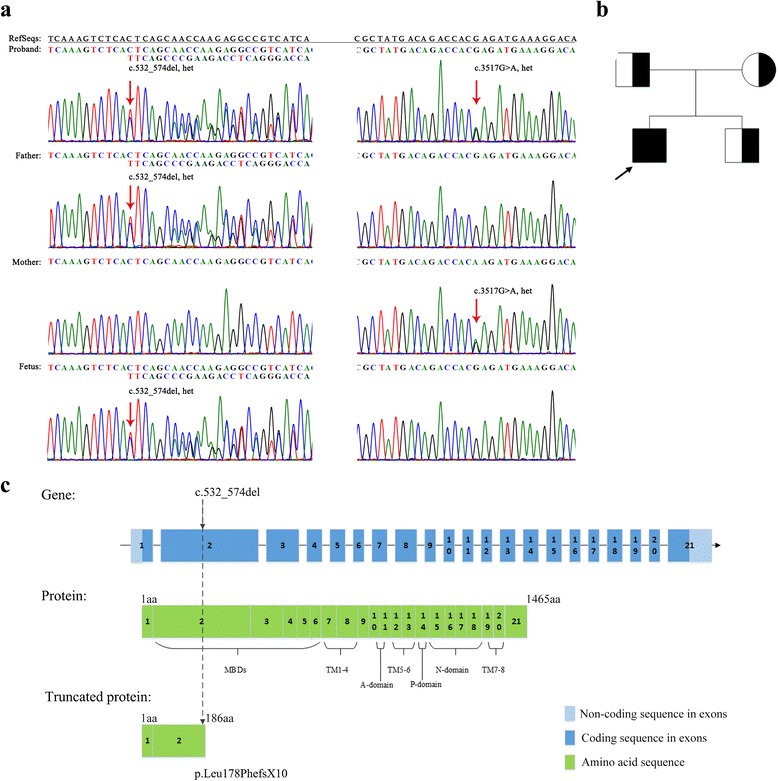
Detection of a novel deletion mutation in the *ATP7B* in a WD patient: c.532_574del (p.Leu178Phefs*10). **a** The compound heterozygous mutation was found in the proband by direct Sanger sequencing. The novel deletion mutation was inherited from his father, and the other reported c.3517G > A (p.Glu1173Lys) mutation was inherited from his mother. The second fetus of the couple had just the heterozygous c.3517G > A mutation. Red arrow, changed base. **b** The pedigree was in line with the typical Mendelian inheritance of autosomal recessive genetic diseases. Black arrow, proband. **c** Illustrative explanation of the deletion mutation affecting ATP7B protein structure and composition. The 43-bp deletion results in a frameshift mutation to produce a truncated protein with 1279 amino-acid residues deleted at the C-terminus, compared with 1465 amino-acid residues in the normal ATP7B protein. Dashed arrows, changed position. MBDs, N-terminal metal-binding domains, including six copper binding domains; TM1–8, eight transmembrane domains building the transmembrane channel for copper transport; A-domain, a phosphatase domain or transduction domain where acyl-phosphate is dephosphorylated, performing ATP hydrolysis for copper cation transport; P-domain, a phosphorylation domain for Asp phosphorylation from the DKTGT sequence; N-domain, an ATP- or nucleotide-binding domain

Sanger direct sequencing of the 21 exons of the *ATP7B* gene revealed two heterozygous mutations in the proband: one missense variant, c.3517G > A (p.Glu1173Lys) and one deletion variant of 43 base pairs, c.532_574del. His mother was heterozygous for the c.3517G > A mutation, while the father was heterozygous for c.532_574del (Fig. 1). Accordingly, the proband was compound heterozygous for c.3517G > A and c.532_574del mutations in both alleles inherited from his mother and father, respectively. The missense mutation p.Glu1173Lys has been reported three times in other cases (Table 1) [18–20]. The c.532_574del mutation was predicted to cause a premature tarnation codon (p.Leu178PhefsX10) in the N-terminal region. According to previous reports and the WD Mutation Database, the c.532_574del mutation in our WD patient was unknown so far. Owing to definite genetic cause of WD in our patient, the parents was offered prenatal diagnosis during their second pregnancy. The fetus inherited the wide-type maternal allele and the deletion-bearing paternal allele. The parents were counseled that the fetus was predicted to be an unaffected carrier of WD.

**Table 1:** *ATP7B* genotypes and phenotypes of WD patients with the c.3517G > A mutation

Patient	Sex	Nationality	Genotype*	Phenotype							references
				Onset age (Y)	K-F ring	ALT (IU/L)	AST (IU/L)	Serum CP (mg/L)	Serum Cu (μmol/L)	Urine Cu (μg/24 h)	
1	M	Chinese	c.532_574del (p.Leu178PhefsX10)	10	+	21	19	20	1.5	256	This study
			c.3517G > A (p.Glu1173Lys)								
2	M	Chinese	c.2333 G > T (p.Arg778Leu)	11	–	162	78	20	\	532	[18]
			c.3517G > A (p.Glu1173Lys)								
3	F	Chinese	c.2975C > T (p.Pro992Leu)	15	+	\	\	45	\	243	[19]
			c.3517G > A (p.Glu1173Lys)								
4	\	France	c.3598C > T(p.Gln1200Ter)	33	\	\	\	120	6.5	\	[20]
			c.3517G > A (p.Glu1173Lys)								

Notes: “-”, negative; “+”, positive; “\”, unknown; “*”, compound heterozygote; *F* female, *M* male, *Y* years, *ALT* alanine aminotransferase, *AST* aspartate aminotransferase, *CP* ceruloplasmin, *Cu* copper. Normal ranges are: ALT, 10~ 40 IU/L; AST, 15~ 46 IU/L; serum CP, 200~ 600 mg/L; serum Cu, 11~ 22 μmol/L; urine Cu, 0~ 60 μg/24 h

The 193 nucleotide sequence including the 43 nucleotide deletion fragment between two breakpoints plus 75 nucleotide fragments surrounding the two breakpoints was used for extensive bioinformatics analysis, assessing the involvement of local genomic architecture in the predisposition to DNA breakage, e.g. repetitive elements, sequence motifs, and non-B DNA conformations, which may initiate the formation of deletions [21]. Known repeats were not found at both breakpoints of the 193-nucleotide sequence by the CENSOR and RepeatMasker tools. Seven sequence motifs previously associated with DNA breakage, including 2 “Deletion hotspot consensus”, 2 “DNA polymerase arrest site”, 1 “Ig heavy chain class switch repeat 1”, 1 “Ig heavy chain class switch repeat 2” and 1 “Vaccinia topoisomerase I consensus”, were observed in the 193-nucleotide sequence using Fuzznuc. No motifs forming left-handed Z-DNA were predicted by nBMST. Direct, inverted, and mirror repeats capable of forming slipped hairpin, cruciform, and triplex structures, respectively, were not found in the above sequence by RepeatAround. The QGRS software revealed oligo(G)n tract forming tetraplex structures in the above nucleotide sequence.

## Discussion and conclusions

The deletion mutation c.532_574del (p.Leu178PhefsX10) in the *ATP7B* gene was reported for the first time in present study, and was observed in proband’s parent, was therefore not a de novo mutation. The 43-bp deletion occurring in exon 2 of the *ATP7B* gene caused a frameshift mutation to yield a truncated ATP7B protein, with 1279 amino-acid residues deleted from the C-terminal segment compared with the mature protein consisting of 1465 amino-acid residues. The ATP7B protein includes five important functional areas that contribute to copper transport, including N-terminal metal-binding, transmembrane, adenosine triphosphate (ATP)-binding, phosphatase, and phosphorylation domains [22]. The deletion mutation removed almost completely these five functional domains (Fig. 1). On the other hand, it resulted in almost 87% amino-acid residues missing from the entire mature protein. The variant would be considered as a pathogenic variant with more severe phenotype than the other missense pathogenic variants. Indeed, the phenotype of the proband with p.Leu178PhefsX10/p.Glu1173Lys compound heterozygote mutations in this study was likely more severe than two reported WD patients whose genotypes were separately p.Pro992Leu/p.Glu1173Lys and p.Gln1200Ter/p.Glu1173Lys (Table 1) [18–20].

After analysis of the c.532_574del mutation in the *ATP7B* gene, two microhomology (DNA direct repeat sequences, TCTCA) were observed on both deletion breakpoints. Based on the short microhomology stretches at breakpoint junctions, a replication-based mechanism named fork stalling and template switching/ microhomology-mediated break-induced replication (FoSTeS/MMBIR) or a non-replicative repair mechanism named microhomology-mediated end-joining (MMEJ) seemed to be able to explain the formation of small deletion mutations [16, 23]. However, MMEJ associated deletions usually have imperfect or interrupted microhomologies at the breakpoints; for instance, distinct 8~ 22 bp pair imperfect microhomologies are frequently observed in the MMEJ events of *S. cerevisiae* and *Drosophila* [10, 24–26]. Meanwhile, MMEJ occasionally generates small scars or causes insertion of nucleotides at the junction site of the deletion in mammalian cells [27, 28]. Indeed, current reports suggest that microhomology is not necessarily essential for MMEJ [28, 29]. MMEJ is currently used to explain the possible formation of large deletions belonging to structural variations (SVs), commonly referred to as copy number variants (CNVs) which are generally defined as DNA regions of approximately 50 bp and larger in size, and not small deletions belonging to indels which are small insertions or deletions generally between 1 and 50 bp in size [25]. The occurrence of FoSTeS/MMBIR events is stimulated by DNA strand breaks in the DNA replication phase. The DNA motifs around the deletion breakpoints would likely cause DNA strand breaks. For example, oligo(G)n tracts have the potential to adopt stable G-quadruplex structures prone to cause genomic alterations or replication fork stalling. Accordingly, the FoSTeS/MMBIR mechanism seemed to better explain the origin of the novel deletion mutation c.532_574del of the *ATP7B* gene in this study.

Several mechanisms of *ATP7B* gene deletions have previously been reported. Alu-mediated MMEJ is a possible mechanism underlying the formation of the 3827 bp deletion in the *ATP7B* gene (c.3134_3556 + 689del) [30]. Meanwhile, Alu-mediated non-allelic homologous recombination (NAHR) seems to favor the formation of the 2776 bp deletion in the *ATP7B* gene (c.52-2461_366del2776) [13]. Therefore, we here report the first case with c.532_574del of the *ATP7B* gene mediated by FoSTeS/MMBIR. Since exactly 1-bp microhomology at the junctions can cause a FoSTeS/MMBIR event, and up to four FoSTeS/MMBIR events occur in the DNA replication phase [14–16], all reported deletion mutations of the *ATP7B* gene were reviewed in Additional file 2: Table S1, and the formation of 65.5% (91/139) small deletions as well as 100% (4/4) gross deletions could be explained by single template switch event in MMBIR using the FoSTeS/MMBIR mechanism; the remaining small deletions could result from two FoSTeS/MMBIR events. Although the FoSTeS/MMBIR mechanism still is imperfect, it provides a perspective to explore the occurrence of deletion mutations in the *ATP7B* gene.

**Fig. 2:**
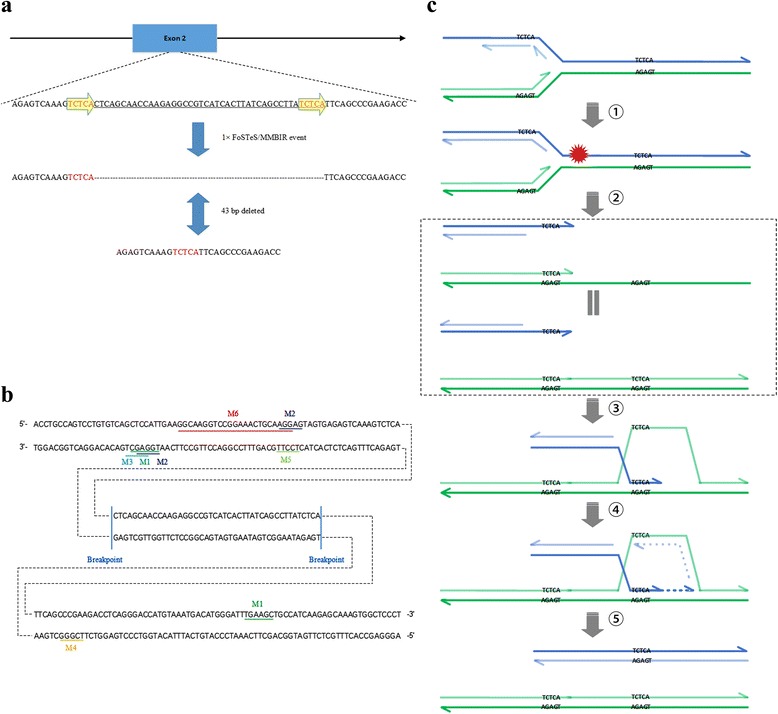
Potential molecular mechanisms underlying the formation process of the c.532_574del mutation in the *ATP7B*. **a** Junction sequence analysis of the 43-bp deletion identified. Five base-pairs of microhomology sequence (TCTCA) were found at both breakpoints. Sequencing revealed that one FoSTeS/MMBIR event caused the deletion during two microhomologies. **b** The presence of sequence motifs, non-B DNA conformations, or repetitive elements increased susceptibility to DNA breakage or promoted replication fork stalling. M1, deletion hotspot consensus (TGRRKM), green underline; M2, DNA polymerase arrest site (WGGAG), dark blue underline; M3, Ig heavy chain class switch repeat 1 (GAGCT), light blue underline; M4, Ig heavy chain class switch repeat 2 (GGGCT), orange underline; M5: Vaccinia topoisomerase I consensus (YCCTT), light green underline; M6, oligo(G)n tracts, red underline. **c** Illustrative explanation of the formation process of the c.532_574del mutation in the ATP7B gene based on FoSTeS/MMBIR mechanism during DNA replication. ① When a replication fork encounters a nick (Red hexagonal star) in a template strand (Blue), an arm of the fork breaks off, producing a collapsed fork. ② At the double-strand end, the 5′ strand (Light blue) is resected, yielding a 3′ overhang (Blue) with the microhomology sequence TCTCA at the terminus. ③ The 3′ single-strand end (Blue) invades the sister chromatid (Green and Light green) in the wrong way so that the microhomology sequence TCTCA (Blue) binds to the base complementary pairing sequence AGAGT of a single strand (Green) of the sister chromatid in the back rather than in the front, forming a D-loop. ④ New replication fork, with both leading and lagging strand replication, was reformed. ⑤ A Holliday junction was present at the site of the D-loop. Migration of the Holliday junction or other helicase activity, separating the extended double-strand end from its templates (Green and Light green). Finally, the replication fork becomes fully mature and continues replication to the chromosome end, which leads to the generation of the c.532_574del mutation. Each line represents a DNA nucleotide chain (strand). Polarity is indicated by half arrows on 3′ end

Published studies indicated that FoSTeS/MMBIR is a mitotic event rather than a meiotic one, e.g. somatic mutations associated with the *PMP22* and *FMR1* genes [16, 31]. Therefore, c.532_574del in the *ATP7B* gene could have occurred through a single FoSTeS/MMBIR event during the mitotic cell division of somatic cells in grandparents or ancestors of the proband (Fig. 2).

In this study, we reported a WD patient with a novel deletion mutation (c.532_574del43) and a known missense mutation (c.3517G > A) located in the two different *ATP7B* alleles. Subsequently, genetic counseling was provided to the proband’s family; prenatal diagnosis for the second pregnancy of his mother was performed using the amniotic fluid.

## Additional file

Additional file 1: Materials and methods. A detailed description of the sample acquisition, sample preparations, Sanger sequencing, alignment, and bioinformatics analyses (DOCX 30 kb)

Additional file 2: **Table S1.** Overview of previously reported pathogenic deletions of the *ATP7B* gene and use of the FoSTeS/MMBIR mechanism to explain their formations (DOCX 149 kb)

## Abbreviations

ATP7B: ATPase copper transporting beta
FoSTeS/MMBIR: fork stalling and template switching/microhomology-mediated break-induced replication
HGMD: Human Gene Mutation Database
HGVS: Human Genome Variation Society
MLPA: Multiples ligation-dependent probe amplification
NCBI: National Center for Biotechnology Information
Nucleotide symbol: A, adenosine; C, cytidine; G, guanosine; T, thymidine
Pairs: K = G/T; M = A/C; R = A/G; S=C/G; W = A/T; Y=C/T. Triples B=C/G/T; D = A/G/T; H = A/C/T; V = A/C/G; N = A/C/G/T
PCR: Polymerase chain reaction
QPCR: quantitative polymerase chain reaction
WD: Wilson’s disease
