# Supplementary material for: Identification and characterization of a novel 43-bp deletion mutation of the ATP7B gene in a Chinese patient with Wilson’s disease: a case report
Source: BMC Med Genet. 2018 Apr 12;19:61. doi: 10.1186/s12881-018-0567-z (PMC5898064; doi:10.1186/s12881-018-0567-z)
Supplement: Supplementary file 1 — Materials and methods. A detailed description of the sample acquisition, sample preparations, Sanger sequencing, alignment, and bioinformatics analyses (DOCX 30 kb) [file 12881_2018_567_MOESM1_ESM.docx]

**Materials and methods**

**Subjects**

The proband was a 10 years old boy diagnosed with Wilson’s disease based on Kayser-Fleischer (KF) rings in the cornea and typical biochemical findings, in the Department of Pediatrics. He was then referred to the Department of Prenatal Diagnosis for molecular diagnosis. Written informed consent for molecular genetic analysis of the ATP7B gene was obtained from the subjects (proband and parents), in this study approved by the Ethics Committee of Nanjing Maternity and Child Health Care Hospital.

**Blood collection and genomic DNA preparation**

About 4-5 mL peripheral blood samples were collected in EDTA anticoagulant tubes from the proband and his parents. Genomic DNA was prepared from blood specimens with an Automated Nucleic Acid Extraction Midi Kit (Zeesan, Xiamen, China) according to the manufacturer’s instructions; the purified DNA was stored in TE buffer (10 mM Tris-Cl, 0.5 mM EDTA, pH 9.0) for downstream applications.

**Genetic analysis by Sanger sequencing**

The coding region spanning 21 exons and the splice sites of 20 introns in the ATP7B gene was PCR amplified using the twenty primer pairs (Table 1). The PCR protocol in this study was slightly modified. Briefly, 22 PCR amplifications were performed in 50μL reactions composed of 1×Ex Taq Buffer, 0.2 mM of each dNTP, 2 mM of Mg2+, 1.25 unit of Ex Taq DNA polymerase (TaKaRa, Dalian, China), 100 ng of DNA template, and 0.4μM of each primer (forward and reverse). PCR was carried out with initial denaturation at 95℃ for 5 minutes, followed by 35 cycles of denaturation (95℃ for 30s), annealing (60℃ for 30s) and extension (72℃ for 45s), and a final extension step (72℃ for 7 min). Then, PCR products were detected by 2% agarose gel electrophoresis; DNA purification of the products was performed with TIANquick Midi Purification Kit (Tiangen, Beijing, China). Subsequently, bidirectional sequencing of various PCR products was performed using an ABI PRISM BigDye 3.1 sequencing kit on an ABI 3130 Genetic Analyzer (Applied Biosystems, Foster City, CA, USA) according to the manufacturer’s instructions. Sanger sequences were analyzed by the SeqMan program within the DNASTAR 7.1 software, and Mutation Surveyor 2.2 was used for comparison with the NCBI reference sequence NG_008806.1. The mutations obtained were described according to the Human Genome Variation Society (HGVS) guidelines (http://www.hgvs.org/mutnomen) and the published RefSeq NM_000053.3 from National Center for Biotechnology Information (NCBI, http://www.ncbi.nlm.nih.gov/).

Table 1. Primers used in the detection for coding sequences of ATP7B gene

| **Exon^a^** | **Primer name** | **Sequence (5′-3′)** | **Tm^b^** | **Product length^c^** |
| --- | --- | --- | --- | --- |
| 1 | E1F | TTGAATCATCCGTGTGAAGAGG | 60℃ | 592bp |
|  | E1R | ACTTCTCCAGGTAACTAACTTCATC |  |  |
| 2 | E2AF | TTGACACCAGTGGCATTGTT | 60℃ | 759bp |
|  | E2AR | GTCTCTTTGGGTTAGTGCTTTG |  |  |
|  | E2BF | CACTTATCAGCCTTATCTCATTCAG | 60℃ | 772 bp |
|  | E2BR | AAGGAGACAAGCTCAGGACAT |  |  |
| 3 | E3F | GCTGAGGGACAAGGTAGTTACT | 60℃ | 573bp |
|  | E3R | GCCAGTTATACAAGGACATTAGACA |  |  |
| 4 | E4F | TAAGAGACCAGACATCGTGATTG | 60℃ | 524bp |
|  | E4R | AAGTCATTGTTGTCGGCTTCA |  |  |
| 5 | E5F | TGCCATCTGCTTCACGATTG | 60℃ | 633bp |
|  | E5R | TTCCTCATCTTTCTCTTACCCATTC |  |  |
| 6 | E6F | GGTATCTGGGATTCCTTCACTC | 60℃ | 584bp |
|  | E6R | TGACTAGCGGCTATCACACT |  |  |
| 7 | E7F | TCACTTGCCTCACCCGTAAA | 60℃ | 771bp |
|  | E7R | GAGACACAGAGTTCACCACAC |  |  |
| 8 | E8F | AATGGAATGCCTTGTGCTGTC | 60℃ | 625bp |
|  | E8R | TGGTGTTCAGAGGAAGTGAGATT |  |  |
| 9 | E9F | TGTCTCTAACACCACGCTTGT | 60℃ | 526bp |
|  | E9R | CTCTGCCTGTCTGAATGTGTATC |  |  |
| 10&11 | E1011F | TCTACCACAGAACTTGTCTTCATG | 60℃ | 715bp |
|  | E1011R | CTGATTTCCCAGAACTCTTCACATA |  |  |
| 12 | E12F | AGCCATGCCAGTCACTAAGA | 60℃ | 754bp |
|  | E12R | GCATTACAACTGAGCACCAATT |  |  |
| 13 | E13F | CTCTGCTCCTGTAATGCCTCT | 60℃ | 598bp |
|  | E13R | GCTACTCTGTTGCTACTGTTGTT |  |  |
| 14 | E14F | TGCTGTGCCATCTCCTGTA | 60℃ | 678bp |
|  | E14R | AATGCCTGTGACACTGAACTC |  |  |
| 15 | E15F | TTCCAGTCGGTAACCTGTTCA | 60℃ | 678bp |
|  | E15R | TTAAGGCAGCCATAAGCAACAA |  |  |
| 16 | E16F | GAGGTGCTTACAAGGTTACAGTT | 60℃ | 536bp |
|  | E16R | GCAATGATGATGACACAGACAGT |  |  |
| 17 | E17F | GGGAGCCACTGCGAAGATT | 60℃ | 627bp |
|  | E17R | AAGACTGGGAAACGGTTAGAGAA |  |  |
| 18&19 | E1819F | GGTTGACCAACATCACTGACTG | 60℃ | 770bp |
|  | E1819R | ATACAGCCAAGCATCTCCACTA |  |  |
| 20 | E20F | GGACTGTGGCTACAGGATGG | 60℃ | 654bp |
|  | E20R | CTGCTCATGGTGCTGATAAGTT |  |  |
| 21 | E21F | AAGTTAGTGGCAGCCTACAATG | 60℃ | 781bp |
|  | E21R | CGCCTCTCACCTTCTACAGT |  |  |

Notes: ^a^ Based on Reference Sequence, NM_000053.3; ^b^ Tm, the melting temperature of the PCR product; ^c^ Based on Reference Sequence, NG_007107.2.

**Bioinformatics analyses of breakpoint regions**

The 193 nucleotide sequence including the 43 nucleotide deletion fragment between two breakpoints plus 75 nucleotide fragments surrounding the two breakpoints was used for extensive bioinformatics analysis, assessing the involvement of local genomic architecture in the predisposition to DNA breakage, e.g. repetitive elements, sequence motifs, and non-B DNA conformations, which may initiate the formation of deletions [[1](#_ENREF_1)]. Presence of repetitive elements at the breakpoint was assessed by CENSOR at http://www.girinst.org/censor/index.php [[2](#_ENREF_2)] and the RepeatMasker software (A.F.A. Smit, R. Hubley & P. Green, unpublished data; Current Version, open-4.0.6) at http://www.repeatmasker.org/cgi-bin/WEBRepeatMasker. A total of 40 different sequence motifs previously associated with DNA breakage were assessed by the Fuzznuc program (http://emboss.bioinformatics.nl/cgi-bin/emboss/fuzznuc?_pref_hide_optional=0) [[3](#_ENREF_3), [4](#_ENREF_4)]. Z-DNA motifs, which are sequences with the propensity to form Z-form DNA, were assessed by nBMST (https://nonb-abcc.ncifcrf.gov/apps/nBMST/default/) [[5](#_ENREF_5)]. RepeatAround in the default parameters (Motive length 8~30) was used to identify direct, inverted, and mirror repeats, which form slipped hairpin, cruciform, and triplex structures, respectively [[6](#_ENREF_6)]. QGRS was used for nucleotide sequence analysis to detect oligo(G)n tracts forming tetraplex structures [[7](#_ENREF_7)].

**References**

1. Verdin H, D'Haene B, Beysen D, Novikova Y, Menten B, Sante T, Lapunzina P, Nevado J, Carvalho CM, Lupski JR *et al*: Microhomology-mediated mechanisms underlie non-recurrent disease-causing microdeletions of the FOXL2 gene or its regulatory domain. *PLoS genetics* 2013, 9(3):e1003358.

2. Kohany O, Gentles AJ, Hankus L, Jurka J: Annotation, submission and screening of repetitive elements in Repbase: RepbaseSubmitter and Censor. *BMC bioinformatics* 2006, 7:474.

3. Abeysinghe SS, Chuzhanova N, Krawczak M, Ball EV, Cooper DN: Translocation and gross deletion breakpoints in human inherited disease and cancer I: Nucleotide composition and recombination-associated motifs. *Human mutation* 2003, 22(3):229-244.

4. Rice P, Longden I, Bleasby A: EMBOSS: the European Molecular Biology Open Software Suite. *Trends in genetics : TIG* 2000, 16(6):276-277.

5. Cer RZ, Donohue DE, Mudunuri US, Temiz NA, Loss MA, Starner NJ, Halusa GN, Volfovsky N, Yi M, Luke BT *et al*: Non-B DB v2.0: a database of predicted non-B DNA-forming motifs and its associated tools. *Nucleic acids research* 2013, 41(Database issue):D94-D100.

6. Goios A, Meirinhos J, Rocha R, Lopes R, Amorim A, Pereira L: RepeatAround: a software tool for finding and visualizing repeats in circular genomes and its application to a human mtDNA database. *Mitochondrion* 2006, 6(4):218-224.

7. Kikin O, D'Antonio L, Bagga PS: QGRS Mapper: a web-based server for predicting G-quadruplexes in nucleotide sequences. *Nucleic acids research* 2006, 34(Web Server issue):W676-682.
