# Supplementary material for: Identification and characterization of a novel 43-bp deletion mutation of the ATP7B gene in a Chinese patient with Wilson’s disease: a case report
Source: BMC Med Genet. 2018 Apr 12;19:61. doi: 10.1186/s12881-018-0567-z (PMC5898064; doi:10.1186/s12881-018-0567-z)
Supplement: Supplementary file 2 — Table S1. Overview of previously reported pathogenic deletions of the ATP7B gene and use of the FoSTeS/MMBIR mechanism to explain their formations (DOCX 149 kb) [file 12881_2018_567_MOESM2_ESM.docx]

**Table S1.** Overview of previously reported *ATP7B* deletions due to FoSTeS/MMBIR mechanism

| Serial number | Name of deletion^a^ | Number of missing bases (bp) | Classification of variant^b^ | explained by FoSTeS/MMBIR | Number of FoSTeS event^c^ | Microhomology^d^ | Source |
| --- | --- | --- | --- | --- | --- | --- | --- |
| 1 | c.-439_-425del | 15 | Indels | Yes | 1× | CG | [[1](#_ENREF_1)] |
| 2 | c.19_20del | 2 | Indels | Yes | 1× | A | [[2](#_ENREF_2)] |
| 3 | c.52-2461_366del | 2776 | SV | Yes | 2× | CT, GCCAG | [[3](#_ENREF_3)] |
| 4 | c.213_214del | 2 | Indels | Yes | 2× | GTC, GTG | [[4](#_ENREF_4)] |
| 5 | c.330del | 1 | Indels | Yes | 1× | A | [[5](#_ENREF_5)] |
| 6 | c.379del | 1 | Indels | Yes | 2× | GCA, AAG | [[6](#_ENREF_6)] |
| 7 | c.383del | 1 | Indels | Yes | 1× | G | Database^e^ |
| 8 | c.397del | 1 | Indels | Yes | 2× | TCC, GG | [[7](#_ENREF_7)] |
| 9 | c.436_1708-953del | 8798 | SV | Yes | 1× | GGTC | [[8](#_ENREF_8)] |
| 10 | c.448_452del | 5 | Indels | Yes | 1× | G | [[9](#_ENREF_9)] |
| 11 | c.453del | 1 | Indels | Yes | 2× | GGG, ATG | [[10](#_ENREF_10)] |
| 12 | c.507del | 1 | Indels | Yes | 1× | A | [[5](#_ENREF_5)] |
| 13 | c.524_525del | 2 | Indels | Yes | 1× | A | Database |
| 14 | c.525_526del | 2 | Indels | Yes | 2× | AA, TG | [[11](#_ENREF_11)] |
| 15 | c.559del | 1 | Indels | Yes | 1× | T | [[12](#_ENREF_12)] |
| 16 | c.561_563del | 3 | Indels | Yes | 1× | A | [[13](#_ENREF_13)] |
| 17 | c.571_574del | 5 | Indels | Yes | 2× | AT, TT | [[14](#_ENREF_14)] |
| 18 | c.643del | 1 | Indels | Yes | 1× | G | [[12](#_ENREF_12)] |
| 19 | c.654_655del | 2 | Indels | Yes | 2× | AAG, TGG | [[15](#_ENREF_15)] |
| 20 | c.678del | 1 | Indels | Yes | 1× | G | [[16](#_ENREF_16)] |
| 21 | c.775del | 1 | Indels | Yes | 1× | C | [[17](#_ENREF_17)] |
| 22 | c.802_808del | 7 | Indels | Yes | 1× | A | [[18](#_ENREF_18)] |
| 23 | c.813del | 1 | Indels | Yes | 2× | TG, GTC | [[19](#_ENREF_19)] |
| 24 | c.845del | 1 | Indels | Yes | 2× | GC, CC | [[20](#_ENREF_20)] |
| 25 | c.847del | 1 | Indels | Yes | 1× | C | [[21](#_ENREF_21)] |
| 26 | c.892del | 1 | Indels | Yes | 1× | C | Database |
| 27 | c.918_931del | 14 | Indels | Yes | 1× | C | [[22](#_ENREF_22)] |
| 28 | c.1073del | 1 | Indels | Yes | 2× | AT, CA | [[22](#_ENREF_22)] |
| 29 | c.1136del | 1 | Indels | Yes | 1× | G | [[23](#_ENREF_23)] |
| 30 | c.1145_1151del | 7 | Indels | Yes | 1× | CT | [[11](#_ENREF_11)] |
| 31 | c.1218del | 1 | Indels | Yes | 2× | TC, GT | [[24](#_ENREF_24)] |
| 32 | c.1292_1293del | 2 | Indels | Yes | 1× | T | [[25](#_ENREF_25)] |
| 33 | c.1340_1343del | 4 | Indels | Yes | 1× | C | [[20](#_ENREF_20)] |
| 34 | c.1384_1400del | 17 | Indels | Yes | 1× | GGCTCCC | [[26](#_ENREF_26)] |
| 35 | c.1436del | 1 | Indels | Yes | 1× | C | [[27](#_ENREF_27)] |
| 36 | c.1449_1456del | 8 | Indels | Yes | 2× | CCA, GCA | [[19](#_ENREF_19)] |
| 37 | c.1470del | 1 | Indels | Yes | 2× | TG, TT | [[12](#_ENREF_12)] |
| 38 | c.1494del | 1 | Indels | Yes | 1× | C | [[12](#_ENREF_12)] |
| 39 | c.1517_1518del | 2 | Indels | Yes | 1× | A | [[28](#_ENREF_28)] |
| 40 | c.1518_1522del | 5 | Indels | Yes | 2× | AT, GG | [[29](#_ENREF_29)] |
| 41 | c.1520_1523del | 4 | Indels | Yes | 1× | AG | [[11](#_ENREF_11)] |
| 42 | c.1636_1637del | 2 | Indels | Yes | 2× | TC, C | [[30](#_ENREF_30)] |
| 43 | c.1639del | 1 | Indels | Yes | 1× | C | [[31](#_ENREF_31)] |
| 44 | c.1648_1654del | 7 | Indels | Yes | 1× | TG | [[32](#_ENREF_32)] |
| 45 | c.1672_1673del | 2 | Indels | Yes | 2× | GAG, CT | [[23](#_ENREF_23)] |
| 46 | c.1705_1707+8del | 11 | Indels | Yes | 2× | CTG, TG | [[11](#_ENREF_11)] |
| 47 | c.1705_1707+10del | 13 | Indels | Yes | 1× | CTG | [[33](#_ENREF_33)] |
| 48 | c.1708-?_1946+?del | ？ | SV | ? | ? | ? | [[34](#_ENREF_34)] |
| 49 | c.1716del | 1 | Indels | Yes | 1× | G | [[16](#_ENREF_16)] |
| 50 | c.1739del | 1 | Indels | Yes | 2× | CC, C | [[35](#_ENREF_35)] |
| 51 | c.1745_1746del | 2 | Indels | Yes | 1× | A | [[21](#_ENREF_21)] |
| 52 | c.1748_1749del | 2 | Indels | Yes | 1× | AG | [[36](#_ENREF_36)] |
| 53 | c.1766del | 1 | Indels | Yes | 2× | GGA, AA | [[12](#_ENREF_12)] |
| 54 | c.1782del | 1 | Indels | Yes | 2× | TA, GC | [[4](#_ENREF_4)] |
| 55 | c.1803del | 1 | Indels | Yes | 1× | C | [[28](#_ENREF_28)] |
| 56 | c.1823_1825del | 3 | Indels | Yes | 2× | GT, A | [[23](#_ENREF_23)] |
| 57 | c.1849del | 1 | Indels | Yes | 1× | G | [[35](#_ENREF_35)] |
| 58 | c.1869+5_8del | 4 | Indels | Yes | 1× | GTAA | [[16](#_ENREF_16)] |
| 59 | c.1870_1871del | 2 | Indels | Yes | 2× | AAG, AA | [[37](#_ENREF_37)] |
| 60 | c.1870-45_2355+189del3837 | 3837 | SV | Yes | 1× | CCA | [[38](#_ENREF_38)] |
| 61 | c.1883_1884del | 2 | Indels | Yes | 2× | C, GC | [[4](#_ENREF_4)] |
| 62 | c.1947del | 1 | Indels | Yes | 1× | G | [[39](#_ENREF_39)] |
| 63 | c.1963del | 1 | Indels | Yes | 1× | C | [[40](#_ENREF_40)] |
| 64 | c.2004_2006del | 3 | Indels | Yes | 1× | AT | [[25](#_ENREF_25)] |
| 65 | c.2008_2013del | 6 | Indels | Yes | 2× | TC, CTG | [[18](#_ENREF_18)] |
| 66 | c.2009_2015del | 7 | Indels | Yes | 1× | CT | [[41](#_ENREF_41)] |
| 67 | c.2018_2030del | 13 | Indels | Yes | 1× | GA | [[42](#_ENREF_42)] |
| 68 | c.2035del | 1 | Indels | Yes | 1× | C | [[36](#_ENREF_36)] |
| 69 | c.2060_2062del | 3 | Indels | Yes | 1× | ACA | [[43](#_ENREF_43)] |
| 70 | c.2097_2099del | 3 | Indels | Yes | 1× | CTT | [[28](#_ENREF_28)] |
| 71 | c.2101_2102del | 2 | Indels | Yes | 1× | T | [[44](#_ENREF_44)] |
| 72 | c.2116_2117del | 2 | Indels | Yes | 1× | T | [[45](#_ENREF_45)] |
| 73 | c.2160del | 1 | Indels | Yes | 1× | A | [[21](#_ENREF_21)] |
| 74 | c.2203_2231del | 29 | Indels | Yes | 1× | TC | [[25](#_ENREF_25)] |
| 75 | c.2227del | 1 | Indels | Yes | 1× | T | [[16](#_ENREF_16)] |
| 76 | c.2280_2301del | 22 | Indels | Yes | 1× | GCCC | [[11](#_ENREF_11)] |
| 77 | c.2292_2312del | 21 | Indels | Yes | 1× | TCTT | [[46](#_ENREF_46)] |
| 78 | c.2304del | 1 | Indels | Yes | 1× | C | [[47](#_ENREF_47)] |
| 79 | c.2364del | 1 | Indels | Yes | 1× | C | [[45](#_ENREF_45)] |
| 80 | c.2391_2393del | 3 | Indels | Yes | 2× | TC, CC | [[48](#_ENREF_48)] |
| 81 | c.2450del | 1 | Indels | Yes | 2× | GGG, GGA | [[49](#_ENREF_49)] |
| 82 | c.2463del | 1 | Indels | Yes | 1× | C | [[4](#_ENREF_4)] |
| 83 | c.2510del | 1 | Indels | Yes | 1× | G | [[19](#_ENREF_19)] |
| 84 | c.2513del | 1 | Indels | Yes | 1× | A | [[10](#_ENREF_10)] |
| 85 | c.2532del | 1 | Indels | Yes | 1× | A | [[4](#_ENREF_4)] |
| 86 | c.2583del | 1 | Indels | Yes | 1× | C | [[43](#_ENREF_43)] |
| 87 | c.2604del | 1 | Indels | Yes | 1× | C | [[50](#_ENREF_50)] |
| 88 | c.2630_2656del | 27 | Indels | Yes | 1× | TA | [[51](#_ENREF_51)] |
| 89 | c.2645_2646del | 1 | Indels | Yes | 1× | CT | [[24](#_ENREF_24)] |
| 90 | c.2648_2649del | 2 | Indels | Yes | 1× | TG | [[52](#_ENREF_52)] |
| 91 | c.2650_2652del | 3 | Indels | Yes | 2× | TG, ATTA | [[10](#_ENREF_10)] |
| 92 | c.2659del | 1 | Indels | Yes | 2× | AAA, CTA | [[53](#_ENREF_53)] |
| 94 | c.2697_2723del | 27 | Indels | Yes | 1× | GGCTCAGAT | [[51](#_ENREF_51)] |
| 95 | c.2736_2746del | 11 | Indels | Yes | 1× | C | [[16](#_ENREF_16)] |
| 96 | c.2765_2773del | 9 | Indels | Yes | 1× | T | [[54](#_ENREF_54)] |
| 97 | c.2806_2808del | 3 | Indels | Yes | 1× | G | [[55](#_ENREF_55)] |
| 98 | c.2810del | 1 | Indels | Yes | 2× | G, GG | [[56](#_ENREF_56)] |
| 99 | c.2848_2850del | 3 | Indels | Yes | 1× | GTT | Database |
| 100 | c.2862_2865del | 4 | Indels | Yes | 1× | T | [[48](#_ENREF_48)] |
| 101 | c.2871del | 1 | Indels | Yes | 1× | C | [[25](#_ENREF_25)] |
| 102 | c.2874del | 1 | Indels | Yes | 2× | AA, AAGCA | [[57](#_ENREF_57)] |
| 103 | c.2887del | 1 | Indels | Yes | 1× | C | [[28](#_ENREF_28)] |
| 104 | c.3026_3028del | 3 | Indels | Yes | 1× | TCA | [[58](#_ENREF_58)] |
| 105 | c.3084_3085del | 2 | Indels | Yes | 1× | A | [[11](#_ENREF_11)] |
| 106 | c.3085_3086del | 2 | Indels | Yes | 2× | AG, TG | [[21](#_ENREF_21)] |
| 107 | c.3111del | 1 | Indels | Yes | 1× | C | [[59](#_ENREF_59)] |
| 108 | c.3134_3556+689del | 3827 | SV | Yes | 1× | GC | [[60](#_ENREF_60)] |
| 109 | c.3140del | 1 | Indels | Yes | 2× | GG, TG | [[61](#_ENREF_61)] |
| 110 | c.3147del | 1 | Indels | Yes | 1× | C | [[21](#_ENREF_21)] |
| 111 | c.3309del | 1 | Indels | Yes | 1× | G | [[28](#_ENREF_28)] |
| 112 | c.3321del | 1 | Indels | Yes | 2× | CAG, AA | [[62](#_ENREF_62)] |
| 113 | c.3350_3353del | 4 | Indels | Yes | 1× | G | [[61](#_ENREF_61)] |
| 114 | c.3376del | 1 | Indels | Yes | 2× | GT, A | [[28](#_ENREF_28)] |
| 115 | c.3402del | 1 | Indels | Yes | 1× | C | [[63](#_ENREF_63)] |
| 116 | c.3419del | 1 | Indels | Yes | 2× | CAG, CC | [[45](#_ENREF_45)] |
| 117 | c.3449del | 1 | Indels | Yes | 1× | A | [[11](#_ENREF_11)] |
| 118 | c.3472_3482del | 11 | Indels | Yes | 2× | AC, TT | [[20](#_ENREF_20)] |
| 119 | c.3538del | 1 | Indels | Yes | 2× | CC, TCC | [[64](#_ENREF_64)] |
| 120 | c.3547_3548del | 2 | Indels | Yes | 2× | TG, T | [[12](#_ENREF_12)] |
| 121 | c.3567_3568del | 2 | Indels | Yes | 1× | TG | [[65](#_ENREF_65)] |
| 122 | c.3578del | 1 | Indels | Yes | 2× | CG, AA | [[14](#_ENREF_14)] |
| 123 | c.3627_3630del | 4 | Indels | Yes | 2× | T, AGC | [[21](#_ENREF_21)] |
| 124 | c.3627_3632del | 6 | Indels | Yes | 2× | GCT, CAT | Database |
| 125 | c.3649_3654del | 6 | Indels | Yes | 1× | TG | [[21](#_ENREF_21)] |
| 126 | c.3664del | 1 | Indels | Yes | 1× | G | Database |
| 127 | c.3700-1del | 1 | Indels | Yes | 1× | G | [[28](#_ENREF_28)] |
| 128 | c.3713_3714del | 2 | Indels | Yes | 1× | A | [[66](#_ENREF_66)] |
| 129 | c.3731del | 1 | Indels | Yes | 2× | GC, G | [[5](#_ENREF_5)] |
| 130 | c.3787del | 1 | Indels | Yes | 2× | TC, CC | [[38](#_ENREF_38)] |
| 131 | c.3794_3803del | 10 | Indels | Yes | 1× | ATGG | [[29](#_ENREF_29)] |
| 132 | c.3852_3875del | 24 | Indels | Yes | 1× | TGTGGCCAT | [[4](#_ENREF_4)] |
| 133 | c.3881del | 1 | Indels | Yes | 2× | AGG, AGC | [[43](#_ENREF_43)] |
| 134 | c.3892_3894del | 3 | Indels | Yes | 1× | GTC | [[67](#_ENREF_67)] |
| 135 | c.3895del | 1 | Indels | Yes | 1× | C | [[68](#_ENREF_68)] |
| 136 | c.3903+1del | 1 | Indels | Yes | 2× | GA, TG | [[69](#_ENREF_69)] |
| 137 | c.4006del | 1 | Indels | Yes | 2× | GG, TA | [[70](#_ENREF_70)] |
| 138 | c.4021+87_4125-2del | 2159 | SV | Yes | 1× | CA | [[71](#_ENREF_71)] |
| 139 | c.4022-?_4398+?del | ？ | SV | ？ | ？ | ? | [[71](#_ENREF_71)] |
| 140 | c.4074del | 1 | Indels | Yes | 1× | C | [[21](#_ENREF_21)] |
| 141 | c.4085_4086del | 2 | Indels | Yes | 2× | CT, TCTG | [[72](#_ENREF_72)] |
| 142 | c.4092_4093del | 2 | Indels | Yes | 1× | GT | [[21](#_ENREF_21)] |
| 143 | c.4094_4097del | 4 | Indels | Yes | 1× | TGT | [[28](#_ENREF_28)] |
| 144 | c.4097_4098del | 2 | Indels | Yes | 1× | TG | [[53](#_ENREF_53)] |
| 145 | c.4100_4101del | 2 | Indels | Yes | 1× | G | [[53](#_ENREF_53)] |
| 146 | c.4195del | 1 | Indels | Yes | 1× | C | [[73](#_ENREF_73)] |
| 147 | c.4374_4375del | 2 | Indels | Yes | 2× | ATGG, GG | [[36](#_ENREF_36)] |

Notes: ^a^ reference sequences NG_008806.1 and NM_00053.3 from GenBank were used, and mutation nomenclature was according to the Human Genome Variation Society guidelines.

^b^ Indels are small insertions or deletions generally between 1 and 50 bp in size; structural variation (SV) is generally defined as an altered region of DNA of approximately 50bp or larger in size.

^c^ 1×, one FoSTeS/MMBIR event; 2×, two FoSTeS/MMBIR events.

^d^ Several base sequences represent the possible complementary template microhomologies used to anneal and prime DNA replication at the time of fork stalling and template switching.

^e^ Database: [www.hgmd.cf.ac.uk](http://www.hgmd.cf.ac.uk), <https://www.ncbi.nlm.nih.gov/clinvar/>, and <http://www.wilsondisease.med.ualberta.ca/>.

**References**

1. Loudianos G, Dessi V, Lovicu M, Angius A, Nurchi A, Sturniolo GC, Marcellini M, Zancan L, Bragetti P, Akar N: Further delineation of the molecular pathology of Wilson disease in the Mediterranean population. *Human mutation* 1998, 12(2):89-94.

2. Vrabelova S, Letocha O, Borsky M, Kozak L: Mutation analysis of the ATP7B gene and genotype/phenotype correlation in 227 patients with Wilson disease. *Molecular Genetics and Metabolism* 2005, 86(1):277-285.

3. Mameli E, Lepori MB, Chiappe F, Ranucci G, Di Dato F, Iorio R, Loudianos G: Wilson's disease caused by alternative splicing and Alu exonization due to a homozygous 3039-bp deletion spanning from intron 1 to exon 2 of the ATP7B gene. *Gene* 2015, 569(2):276-279.

4. Figus A, Angius A, Loudianos G, Bertini C, Dessi V, Loi A, Deiana M, Lovicu M, Olla N, Sole G *et al*: Molecular pathology and haplotype analysis of Wilson disease in Mediterranean populations. *Am J Hum Genet* 1995, 57(6):1318-1324.

5. Abdelghaffar TY, Elsayed SM, Elsobky E, Bochow B, Buttner J, Schmidt H: Mutational analysis of ATP7B gene in Egyptian children with Wilson disease: 12 novel mutations. *Journal of human genetics* 2008, 53(8):681-687.

6. Denoyer Y, Woimant F, Bost M, Edan G, Drapier S: Neurological Wilson's disease lethal for the son, asymptomatic in the father. *Mov Disord* 2013, 28(3):402-403.

7. Dedoussis GV, Genschel J, Sialvera TE, Bochow B, Manolaki N, Manios Y, Tsafantakis E, Schmidt H: Wilson disease: high prevalence in a mountainous area of Crete. *Ann Hum Genet* 2005, 69(Pt 3):268-274.

8. Incollu S, Lepori MB, Zappu A, Dessi V, Noli MC, Mameli E, Iorio R, Ranucci G, Cao A, Loudianos G: DNA and RNA studies for molecular characterization of a gross deletion detected in homozygosity in the NH2-terminal region of the ATP7B gene in a Wilson disease patient. *Molecular and cellular probes* 2011, 25(5-6):195-198.

9. Gupta A, Aikath D, Neogi R, Datta S, Basu K, Maity B, Trivedi R, Ray J, Das SK, Gangopadhyay PK *et al*: Molecular pathogenesis of Wilson disease: haplotype analysis, detection of prevalent mutations and genotype-phenotype correlation in Indian patients. *Human genetics* 2005, 118(1):49-57.

10. Okada T, Shiono Y, Hayashi H, Satoh H, Sawada T, Suzuki A, Takeda Y, Yano M, Michitaka K, Onji M *et al*: Mutational analysis of ATP7B and genotype-phenotype correlation in Japanese with Wilson's disease. *Human mutation* 2000, 15(5):454-462.

11. Curtis D, Durkie M, Balac P, Sheard D, Goodeve A, Peake I, Quarrell O, Tanner S: A study of Wilson disease mutations in Britain. *Human mutation* 1999, 14(4):304-311.

12. Coffey AJ, Durkie M, Hague S, McLay K, Emmerson J, Lo C, Klaffke S, Joyce CJ, Dhawan A, Hadzic N *et al*: A genetic study of Wilson's disease in the United Kingdom. *Brain* 2013, 136(Pt 5):1476-1487.

13. Cox DW, Prat L, Walshe JM, Heathcote J, Gaffney D: Twenty-four novel mutations in Wilson disease patients of predominantly European ancestry. *Human mutation* 2005, 26(3):280.

14. Lee BH, Kim JH, Lee SY, Jin HY, Kim KJ, Lee JJ, Park JY, Kim GH, Choi JH, Kim KM *et al*: Distinct clinical courses according to presenting phenotypes and their correlations to ATP7B mutations in a large Wilson's disease cohort. *Liver international : official journal of the International Association for the Study of the Liver* 2011, 31(6):831-839.

15. Chappuis P, Callebert J, Quignon V, Woimant F, Laplanche JL: Late neurological presentations of Wilson disease patients in French population and identification of 8 novel mutations in the ATP7B gene. *J Trace Elem Med Biol* 2007, 21(1):37-42.

16. Aggarwal A, Chandhok G, Todorov T, Parekh S, Tilve S, Zibert A, Bhatt M, Schmidt HH: Wilson disease mutation pattern with genotype-phenotype correlations from Western India: confirmation of p.C271* as a common Indian mutation and identification of 14 novel mutations. *Ann Hum Genet* 2013, 77(4):299-307.

17. Elleuch N, Feki I, Turki E, Miladi MI, Boukhris A, Damak M, Mhiri C, Chappuis E, Woimant F: [A novel mutation in ATP7B gene associated with severe neurological impairment in Wilson's disease]. *Rev Neurol (Paris)* 2010, 166(5):550-552.

18. Haas R, Gutierrez-Rivero B, Knoche J, Boker K, Manns MP, Schmidt HH: Mutation analysis in patients with Wilson disease: identification of 4 novel mutations. Mutation in brief no. 250. Online. *Human mutation* 1999, 14(1):88.

19. Li XH, Lu Y, Ling Y, Fu QC, Xu J, Zang GQ, Zhou F, De-Min Y, Han Y, Zhang DH *et al*: Clinical and molecular characterization of Wilson's disease in China: identification of 14 novel mutations. *BMC Med Genet* 2011, 12:6.

20. Shah AB, Chernov I, Zhang HT, Ross BM, Das K, Lutsenko S, Parano E, Pavone L, Evgrafov O, Ivanova-Smolenskaya IA *et al*: Identification and analysis of mutations in the Wilson disease gene (ATP7B): population frequencies, genotype-phenotype correlation, and functional analyses. *Am J Hum Genet* 1997, 61(2):317-328.

21. Thomas GR, Forbes JR, Roberts EA, Walshe JM, Cox DW: The Wilson disease gene: spectrum of mutations and their consequences. *Nature genetics* 1995, 9(2):210-217.

22. Davies LP, Macintyre G, Cox DW: New mutations in the Wilson disease gene, ATP7B: implications for molecular testing. *Genet Test* 2008, 12(1):139-145.

23. Loudianos G, Dessi V, Lovicu M, Angius A, Nurchi A, Sturniolo GC, Marcellini M, Zancan L, Bragetti P, Akar N *et al*: Further delineation of the molecular pathology of Wilson disease in the Mediterranean population. *Human mutation* 1998, 12(2):89-94.

24. Weiss KH, Runz H, Noe B, Gotthardt DN, Merle U, Ferenci P, Stremmel W, Fullekrug J: Genetic analysis of BIRC4/XIAP as a putative modifier gene of Wilson disease. *J Inherit Metab Dis* 2010, 33 Suppl 3:S233-240.

25. Nanji MS, Nguyen VT, Kawasoe JH, Inui K, Endo F, Nakajima T, Anezaki T, Cox DW: Haplotype and mutation analysis in Japanese patients with Wilson disease. *Am J Hum Genet* 1997, 60(6):1423-1429.

26. Liu XQ, Zhang YF, Liu TT, Hsiao KJ, Zhang JM, Gu XF, Bao KR, Yu LH, Wang MX: Correlation of ATP7B genotype with phenotype in Chinese patients with Wilson disease. *World J Gastroenterol* 2004, 10(4):590-593.

27. Deguti MM, Genschel J, Cancado EL, Barbosa ER, Bochow B, Mucenic M, Porta G, Lochs H, Carrilho FJ, Schmidt HH: Wilson disease: novel mutations in the ATP7B gene and clinical correlation in Brazilian patients. *Human mutation* 2004, 23(4):398.

28. Gu YH, Kodama H, Du SL, Gu QJ, Sun HJ, Ushijima H: Mutation spectrum and polymorphisms in ATP7B identified on direct sequencing of all exons in Chinese Han and Hui ethnic patients with Wilson's disease. *Clin Genet* 2003, 64(6):479-484.

29. Vrabelova S, Letocha O, Borsky M, Kozak L: Mutation analysis of the ATP7B gene and genotype/phenotype correlation in 227 patients with Wilson disease. *Mol Genet Metab* 2005, 86(1-2):277-285.

30. Zali N, Mohebbi SR, Esteghamat S, Chiani M, Haghighi MM, Hosseini-Asl SM, Derakhshan F, Mohammad-Alizadeh AH, Malek-Hosseini SA, Zali MR: Prevalence of ATP7B Gene Mutations in Iranian Patients With Wilson Disease. *Hepat Mon* 2011, 11(11):890-894.

31. Kalinsky H, Funes A, Zeldin A, Pel-Or Y, Korostishevsky M, Gershoni-Baruch R, Farrer LA, Bonne-Tamir B: Novel ATP7B mutations causing Wilson disease in several Israeli ethnic groups. *Human mutation* 1998, 11(2):145-151.

32. Fan Y, Yu L, Han Y, Ren M, Yang R, Zhao S: Identification of three novel insertion/deletion mutations in Wilson disease's gene. *Biochem Genet* 2004, 42(9-10):377-384.

33. Dufernez F, Lachaux A, Chappuis P, De Lumley L, Bost M, Woimant F, Misrahi M, Debray D: Wilson disease in offspring of affected patients: report of four French families. *Clin Res Hepatol Gastroenterol* 2013, 37(3):240-245.

34. Moller LB, Horn N, Jeppesen TD, Vissing J, Wibrand F, Jennum P, Ott P: Clinical presentation and mutations in Danish patients with Wilson disease. *Eur J Hum Genet* 2011, 19(9):935-941.

35. Lepori MB, Zappu A, Incollu S, Dessi V, Mameli E, Demelia L, Nurchi AM, Gheorghe L, Maggiore G, Sciveres M *et al*: Mutation analysis of the ATP7B gene in a new group of Wilson's disease patients: contribution to diagnosis. *Molecular and cellular probes* 2012, 26(4):147-150.

36. Lepori MB, Lovicu M, Dessi V, Zappu A, Incollu S, Zancan L, Giacchino R, Iorio R, Vajro P, Maggiore G *et al*: Twenty-four novel mutations in Wilson disease patients of predominantly Italian origin. *Genet Test* 2007, 11(3):328-332.

37. Treepongkaruna S, Pienvichit P, Phuapradit P, Kodcharin P, Wattanasirichaigoon D: Mutations of ATP7B gene in two Thai siblings with Wilson disease. *Asian Biomedicine* 2010, 4(1):163-169.

38. Tatsumi Y, Shinohara T, Imoto M, Wakusawa S, Yano M, Hayashi K, Hattori A, Hayashi H, Shimizu A, Ichiki T *et al*: Potential of the international scoring system for the diagnosis of Wilson disease to differentiate Japanese patients who need anti-copper treatment. *Hepatol Res* 2011, 41(9):887-896.

39. Tatsumi Y, Hattori A, Hayashi H, Ikoma J, Kaito M, Imoto M, Wakusawa S, Yano M, Hayashi K, Katano Y *et al*: Current state of Wilson disease patients in central Japan. *Intern Med* 2010, 49(9):809-815.

40. Santhosh S, Shaji RV, Eapen CE, Jayanthi V, Malathi S, Chandy M, Stanley M, Selvi S, Kurian G, Chandy GM: ATP7B mutations in families in a predominantly Southern Indian cohort of Wilson's disease patients. *Indian J Gastroenterol* 2006, 25(6):277-282.

41. Thomas GR, Jensson O, Gudmundsson G, Thorsteinsson L, Cox DW: Wilson disease in Iceland: a clinical and genetic study. *Am J Hum Genet* 1995, 56(5):1140-1146.

42. Bem RS, Raskin S, Muzzillo DA, Deguti MM, Cancado EL, Araujo TF, Nakhle MC, Barbosa ER, Munhoz RP, Teive HA: Wilson's disease in Southern Brazil: genotype-phenotype correlation and description of two novel mutations in ATP7B gene. *Arq Neuropsiquiatr* 2013, 71(8):503-507.

43. Mukherjee S, Dutta S, Majumdar S, Biswas T, Jaiswal P, Sengupta M, Bhattacharya A, Gangopadhyay PK, Bavdekar A, Das SK *et al*: Genetic defects in Indian Wilson disease patients and genotype-phenotype correlation. *Parkinsonism Relat Disord* 2014, 20(1):75-81.

44. Loudianos G, Lovicu M, Solinas P, Kanavakis E, Tzetis M, Manolaki N, Panagiotakaki E, Karpathios T, Cao A: Delineation of the spectrum of Wilson disease mutations in the Greek population and the identification of six novel mutations. *Genet Test* 2000, 4(4):399-402.

45. Kumar S, Thapa BR, Kaur G, Prasad R: Identification and molecular characterization of 18 novel mutations in the ATP7B gene from Indian Wilson disease patients: genotype. *Clin Genet* 2005, 67(5):443-445.

46. Eapen CE, Santhosh S, Shaji RV, Chandy M, Chandy GM: Gene symbol: ATP7B. Disease: Wilson's disease. *Human genetics* 2004, 114(6):606.

47. Kim EK, Yoo OJ, Song KY, Yoo HW, Choi SY, Cho SW, Hahn SH: Identification of three novel mutations and a high frequency of the Arg778Leu mutation in Korean patients with Wilson disease. *Human mutation* 1998, 11(4):275-278.

48. Margarit E, Bach V, Gomez D, Bruguera M, Jara P, Queralt R, Ballesta F: Mutation analysis of Wilson disease in the Spanish population -- identification of a prevalent substitution and eight novel mutations in the ATP7B gene. *Clin Genet* 2005, 68(1):61-68.

49. Abdel Ghaffar TY, Elsayed SM, Elnaghy S, Shadeed A, Elsobky ES, Schmidt H: Phenotypic and genetic characterization of a cohort of pediatric Wilson disease patients. *BMC Pediatr* 2011, 11:56.

50. Mak CM, Lam CW, Tam S, Lai CL, Chan LY, Fan ST, Lau YL, Lai ST, Yuen P, Hui J *et al*: Mutational analysis of 65 Wilson disease patients in Hong Kong Chinese: identification of 17 novel mutations and its genetic heterogeneity. *Journal of human genetics* 2008, 53(1):55-63.

51. Yoo HW: Identification of novel mutations and the three most common mutations in the human ATP7B gene of Korean patients with Wilson disease. *Genet Med* 2002, 4(6 Suppl):43S-48S.

52. Loudianos G, Kostic V, Solinas P, Lovicu M, Dessi V, Svetel M, Major T, Cao A: Characterization of the molecular defect in the ATP7B gene in Wilson disease patients from Yugoslavia. *Genet Test* 2003, 7(2):107-112.

53. Yamaguchi A, Matsuura A, Arashima S, Kikuchi Y, Kikuchi K: Mutations of ATP7B gene in Wilson disease in Japan: identification of nine mutations and lack of clear founder effect in a Japanese population. *Human mutation* 1998, Suppl 1:S320-322.

54. Okada T, Morise T, Takeda Y, Mabuchi H: A new variant deletion of a copper-transporting P-type ATPase gene found in patients with Wilson's disease presenting with fulminant hepatic failure. *J Gastroenterol* 2000, 35(4):278-283.

55. Yang J, Chan P: Gene symbol: ATP7B. Disease: Wilson's disease. *Human genetics* 2005, 118(3-4):539.

56. Wu ZY, Wang N, Lin MT, Fang L, Murong SX, Yu L: Mutation analysis and the correlation between genotype and phenotype of Arg778Leu mutation in chinese patients with Wilson disease. *Arch Neurol* 2001, 58(6):971-976.

57. Shimizu N, Kawase C, Nakazono H, Hemmi H, Shimatake H, Aoki T: A novel RNA splicing mutation in Japanese patients with Wilson disease. *Biochem Biophys Res Commun* 1995, 217(1):16-20.

58. Balakrishnan P, Kabra M, Arora NK, Kalra V: Gene symbol: ATP7B. *Human genetics* 2007, 120(6):917.

59. Simsek Papur O, Akman SA, Cakmur R, Terzioglu O: Mutation analysis of ATP7B gene in Turkish Wilson disease patients: identification of five novel mutations. *Eur J Med Genet* 2013, 56(4):175-179.

60. Todorov T, Balakrishnan P, Savov A, Socha P, Schmidt HH: Intragenic Deletions in ATP7B as an Unusual Molecular Genetics Mechanism of Wilson's Disease Pathogenesis. *PLoS One* 2016, 11(12):e0168372.

61. Gromadzka G, Schmidt HH, Genschel J, Bochow B, Rodo M, Tarnacka B, Litwin T, Chabik G, Czlonkowska A: Frameshift and nonsense mutations in the gene for ATPase7B are associated with severe impairment of copper metabolism and with an early clinical manifestation of Wilson's disease. *Clin Genet* 2005, 68(6):524-532.

62. Gojova L, Jansova E, Kulm M, Pouchla S, Kozak L: Genotyping microarray as a novel approach for the detection of ATP7B gene mutations in patients with Wilson disease. *Clin Genet* 2008, 73(5):441-452.

63. Tanzi RE, Petrukhin K, Chernov I, Pellequer JL, Wasco W, Ross B, Romano DM, Parano E, Pavone L, Brzustowicz LM *et al*: The Wilson disease gene is a copper transporting ATPase with homology to the Menkes disease gene. *Nature genetics* 1993, 5(4):344-350.

64. Shimizu N, Nakazono H, Takeshita Y, Ikeda C, Fujii H, Watanabe A, Yamaguchi Y, Hemmi H, Shimatake H, Aoki T: Molecular analysis and diagnosis in Japanese patients with Wilson's disease. *Pediatr Int* 1999, 41(4):409-413.

65. Panichareon B, Taweechue K, Thongnoppakhun W, Aksornworanart M, Pithukpakorn M, Yenchitsomanus PT, Limwongse C, Limjindaporn T: Six novel ATP7B mutations in Thai patients with Wilson disease. *Eur J Med Genet* 2011, 54(2):103-107.

66. Waldenstrom E, Lagerkvist A, Dahlman T, Westermark K, Landegren U: Efficient detection of mutations in Wilson disease by manifold sequencing. *Genomics* 1996, 37(3):303-309.

67. Kusuda Y, Hamaguchi K, Mori T, Shin R, Seike M, Sakata T: Novel mutations of the ATP7B gene in Japanese patients with Wilson disease. *Journal of human genetics* 2000, 45(2):86-91.

68. Lewis PD: Novel human pathological mutations. *Human genetics* 2006, 119:359-364.

69. Duc HH, Hefter H, Stremmel W, Castaneda-Guillot C, Hernandez Hernandez A, Cox DW, Auburger G: His1069Gln and six novel Wilson disease mutations: analysis of relevance for early diagnosis and phenotype. *Eur J Hum Genet* 1998, 6(6):616-623.

70. Li K, Zhang WM, Lin S, Wen L, Wang ZF, Xie D, Wei M, Qiu ZQ, Dai Y, Lin MC *et al*: Mutational analysis of ATP7B in north Chinese patients with Wilson disease. *Journal of human genetics* 2013, 58(2):67-72.

71. Moller LB, Ott P, Lund C, Horn N: Homozygosity for a gross partial gene deletion of the C-terminal end of ATP7B in a Wilson patient with hepatic and no neurological manifestations. *Am J Med Genet A* 2005, 138(4):340-343.

72. Caca K, Ferenci P, Kuhn HJ, Polli C, Willgerodt H, Kunath B, Hermann W, Mossner J, Berr F: High prevalence of the H1069Q mutation in East German patients with Wilson disease: rapid detection of mutations by limited sequencing and phenotype-genotype analysis. *J Hepatol* 2001, 35(5):575-581.

73. Majumdar R, Al Jumah M, Fraser M: 4193delC, a common mutation causing Wilson's disease in Saudi Arabia: rapid molecular screening of patients and carriers. *Mol Pathol* 2003, 56(5):302-304.
